# Anæsthetisation for Operations within the Abdomen

**Published:** 1908-04-11

**Authors:** J. D. Mortimer

**Affiliations:** Anæsthetist to the Royal Waterloo Hospital.


### The General Practitioner's Column.

[Contributions to this Column are invited, and if accepted will be paid for.]

### ANÆSTHETISATION FOR OPERATIONS WITHIN THE ABDOMEN.

By J. D. Mortimer, M.B., F.R.C.S. Anæsthetist to the Royal Waterloo Hospital.

In regard to the choice of an anæsthetic for an abdominal operation, it may be said at once that neither in such cases nor in any others should a "favourite" anæsthetic be given as a matter of routine. One should be guided not only by the age, general condition, state of respiratory and circulatory organs, and other considerations common to all operations, but also by the special necessity for avoidance of rigidity, cough, or vomiting, which are not only inconvenient to the operator, but also dangerous to the patient. It must not be forgotten that operations on the abdominal organs are often prolonged, and that reflex disturbances, sometimes of a serious nature, are prone to occur during the handling of its contents. On the general considerations which should influence the choice of an anæsthetic it is not proposed to dwell here in detail, but as regards infants and children some protest may be entered against a too common belief that even weakly ones can take chloroform with impunity. The young have no special immunity, although in consequence of the absence of degenerative changes they may be more easily rescued than adults from perilous conditions. In debilitated subjects, ether by the open method, a nitrous-oxide-ether or ethyl-chloride-ether sequence, A.C.E. (or C.E.) should be used, at any rate at the commencement. In adolesence and early middle life ether, preceded by nitrous oxide or ethyl chloride is usually taken well during induction; but florid, muscular subjects are apt to struggle much, and become rigid and cyanosed, especially when the upper respiratory passages are irritable from smoking, alcoholism, or otherwise; by these A.C.E. is generally taken comparatively quietly, and time is saved in the end. In later middle life ether is unsuitable for obese and flabby patients. Although induction may be smooth, much mucus is likely to be secreted, and as cough cannot be allowed, cyanosis will occur: A.C.E. is preferable. To the old, chloroform may be given from the first if there is no marked pallor nor weakness. Ether is usually inadmissible on account of rigidity of the chest and degenerative changes in the respiratory and circulatory tracts. If, however, there is much obesity, probable degeneration of the cardiac muscle, or very evident debility, it may be well to start with A.C.E., changing to chloroform later on if such is indicated. In any case where there is extreme abdominal distension, or inactivity of the abdominal muscles, as in acute peritonitis, A.C.E. is preferable to ether. Where there is extreme feebleness or probable shock from any cause, care must be taken to keep the patient warm by cotton-wool packing, hot-water bottles, etc., and materials should be at hand for saline infusion should this be required. In acute intestinal obstruction and the like, where there is persistent vomiting, general anæsthesia cannot be expected to hold this in abeyance; therefore anæsthesia must, contrary to the usual rule in abdominal operations, be light, otherwise there is increased risk that vomited fluid and particles will be inhaled instead of being coughed out. A preliminary washing out of the stomach is advised by some authorities. In many instances, however, this will prove a failure, fluid quickly re-accumulating and being again discharged; the question of preliminary tracheotomy and plugging should be considered, or the employment of local anæsthesia exclusively or in combination. It may be noted that death occurring just after vomiting is by no means always due to suffocation. It may be due to accompanying syncope, or the vomiting may be merely the result of a terminal muscular contraction. Inquiry should be made as to previous administration of opium, which affects the pupil and influences the amount of anæsthetic required; the respiration then needs careful watching to avoid an over-dose.

Many surgeons object to ether, and even to a mixture containing it, in abdominal operations, on the ground that the "coughing, cyanosis with accompanying venous oozing, exaggerated respiratory movements, and rigidity are highly inconvenient." As regards these objections, much depends on a proper selection of cases, and on the way in which the anæsthetic is given. In suitable cases, the period of induction is quiet, and coughing and choking should not occur afterwards. Cyanosis and laboured breathing should be avoided by not employing the "asphyxial element"—if the "open" method be not employed, the apparatus should be often removed from the face, and a free air-way always maintained, the lower jaw being pushed forward if necessary. Rigidity may be the result of a neglect of these precautions, but it may be due to a light anæsthesia; or to contraction of the abdominal muscles dependent on underlying acute disease, or, even in deep anæsthesia, to interference with sensitive parts. In the last two instances relaxation may often be procured by the temporary substitution of chloroform, given cautiously on account of the previous stimulation by ether; sometimes only very little is needed, and ether or a mixture may then be resumed without inconvenience. An early change to chloroform should however be made if over-activity of the circulation and breathing seems likely to cause any difficulty to the operator.

In the Trendelenburg position chloroform may be given without hesitation in many cases where one would otherwise prefer not to do so. It is seldom admissible in old people, and is contra-indicated by many conditions, such as obesity, plethora, and defective respiration.

It must not be forgotten that some of the dangers of chloroform will be avoided by not giving it during the period of induction, and also that reflex cardiac depression from manipulation of sensitive structures is unquestionably more apt to occur, and is more likely to be serious, when a patient is taking chloroform alone than when ether (or an ether mixture) is employed. For this reason some operators prefer the use of the more stimulating anæsthetic throughout.

During the operation, the anæsthetist should notice what the surgeon and his assistants are doing, but mainly in order that he may regulate the depth of anæsthesia accordingly, and may be on the watch for possible results, such as shock or vomiting; he must not allow his interest in surgical details to divert his attention from his own duties. This is one reason why it is better for the practitioner in attendance on a patient not to give the anæsthetic.

Fluid may escape very quietly from the stomach if direct or indirect pressure is made upon it. It is hardly necessary to say that a wedge, gag, sponges, etc., should be always at hand, and that on its occurrence the head must be turned well to the side. It is better not to draw the tongue forward until the pharynx is cleared, as the risk of inhalation of fluid is increased by doing so.

Constant attention is necessary to maintain a level of anæsthesia deep enough for the purposes of the operator, but not so deep as to endanger the patient's safety. This is often difficult with those whose nervous systems are so sensitive that reflex disturbances are easily produced, and with those who are found to require less than the average amount of the anæsthetic; these are apt to pass quickly from a profound anæsthesia to one which is too light for the occasion.

Anæsthesia should be kept up until the application of the bandage. Some surgeons spend much more time than others in suturing the parietes, and there is always a possibility of delay in the completion of the operation occurring from various causes. Some movement of the patient in the final toilet is occasionally needed, and if premature coughing or vomiting occur much mischief may be done. The bandage should not be so applied that the respiration is likely to be seriously hampered, especially when this function is already impaired. It is not advisable to give a morphine injection until the patient begins to "come round" and has coughed up any mucus that may be present in the air-passages.

It is hoped that the foregoing article may be helpful to many, but the writer feels bound to add that not only during operations in the abdomen, but also in many other regions, success in the administration of the anæsthetic mainly depends upon practical experience; and that those who have not had opportunities of acquiring such under skilled supervision should not, except in cases of emergency, undertake such a heavy responsibility.

